# Gene variant analysis in pediatrics with early-onset epilepsy: Identification of novel variants

**DOI:** 10.1016/j.plabm.2025.e00462

**Published:** 2025-03-19

**Authors:** Pooyan Alizadeh, Armin Jahangiri Babadi, Nemat Ghadiri, Mostafa Neissi, Masoud Zeinali

**Affiliations:** a Department of Neurosurgery, Faculty of Medicine, Ahvaz Jundishapur University of Medical Sciences, Ahvaz, Iran; b Department of Genetics, Khuzestan Science and Research Branch, Islamic Azad University, Ahvaz, Iran; c Department of Genetics, Ahvaz Branch, Islamic Azad University, Ahvaz, Iran; d Noor-Gene Genetic Laboratory, Ahvaz, Iran

**Keywords:** Epilepsy, Whole-exome sequencing, Pediatric epilepsy, Genetic analysis

## Abstract

**Background:**

Epilepsy encompasses a range of brain disorders, often accompanied by growth delay and cerebral palsy. The identification of gene variants is critical for guiding treatment strategies in patients with epilepsy. This study investigates the genetic variants in patients with early-onset epilepsy (EOE) through whole-exome sequencing (WES).

**Materials and methods:**

DNA was extracted from peripheral blood using a standard salting-out method. Gene variants were identified using WES, and sequencing data were analyzed through a two-step approach.

**Results:**

Among 20 subjects, WES identified two novel variants. The first variant, **AP3B2** (NM_001278512.2: c.3190G > A; p. Val1064Ile), was located in exon 27 and exhibited homozygosity in the proband and heterozygosity in the parents. The second variant, **PIGB** (NM_004855.5: c.1664G > C; p.Ter555Serext∗54), was located in exon 12 and demonstrated a similar inheritance pattern. Notably, the **PIGB** variant was associated with elevated ALP levels.

**Conclusion:**

This study highlights the value of WES in identifying genetic variants associated with epilepsy, particularly the novel AP3B2 and PIGB variants. By focusing on these impactful findings, the study advances understanding of epilepsy genetics and emphasizes the role of WES in enabling early diagnosis, personalized treatment, and improved management strategies.

## 1 Background

One of the most challenging aspects of epilepsy management is increasing the response rate to its treatment. According to studies, it has been shown that there is a significant gap in the treatment of epilepsy in different countries [1]. Among the things that affect the treatment of epilepsy is its pathogenesis, especially in genetic cases.

Epilepsy is one of the disorders that is highly influenced by genetics. Gene variants in early neural development cause functional and structural impairment, resulting in seizures. Gene variants determine the threshold of seizure [2,3]. In addition to inheritance, epilepsy can be due to gene variants without familial history and clinical findings; however, it presents in the next generations, especially in consanguineous marriages. In this line, next-generation sequencing (NGS) techniques provide insightful findings regarding epilepsy pathophysiology, which is very important in determining the most appropriate treatment strategy. The high number of gene variants involved in the development of monogenic, familial, and encephalopathy epilepsies were identified by NGS methods, such as whole-exome sequencing (WES) [4,5]. It was demonstrated that WES has the highest diagnostic yield among NGS techniques [6].

The type of genetic impairment is directly associated with the response to treatment [[7], [8], [9]]. For this purpose, this study aims to investigate the existence and determine the type of genetic variants in patients with early-onset epilepsy (EOE).

## 2 Material and methods

### 2.1 Study population

In the present retrospective study, patients with EOE were evaluated. Patient selection was conducted among subjects who were referred to the neurology department. The inclusion criteria were as follows: seizures onset less than 3 years old and without history of genetic analysis. Brain tumors, history of just one-time seizure following fever, neurological disorders, secondary epilepsy, and brain trauma were considered as exclusion criteria. Their parents were called for further genetic analysis in cases where gene variants were identified. A thorough review of subjects' medical records was conducted to gather comprehensive clinical data.

**Ethical considerations:** Informed consent was obtained from all adult participants. The current study is based on the ethical committee of Ahvaz Jundishapur University of Medical Sciences (IR.AJUMS.HGOLESTAN.REC.1403.010). All procedures performed in studies involving human participants followed the ethical standards of the institutional and/or national research committee, along with the 1964 Helsinki Declaration and its later amendments or compared ethical strands.

### 2.2 Whole-exome sequencing

WES was exclusively employed for the patients in our study to investigate potential genetic causes of Epileptic Encephalopathy. We focused on a custom panel of 191 genes, selected based on their known or suspected involvement in epilepsy and neurodevelopmental disorders. The selection criteria included genes with strong evidence of association from peer-reviewed literature, databases such as OMIM (https://www.omim.org/), ClinVar (https://www.ncbi.nlm.nih.gov/clinvar/), HGMD (https://www.hgmd.cf.ac.uk/ac/index.php), GeneReviews [10], and professional guidelines for epilepsy and neurogenetics. This panel incorporated genes associated with monogenic epilepsy syndromes, metabolic epilepsies, ion channelopathies, and other neurodevelopmental conditions linked to EOE. To further assess variants' pathogenicity, we cross-referenced them with population databases, including gnomAD and the 1000 Genomes Project. Variants with a frequency higher than 1 % were filtered out, and only rare or low-frequency variants (below 1 %) were considered. Hence, any variant diagnosed by this method can be considered extremely rare or absent in the general population, further supporting its potential pathogenicity. DNA extraction from peripheral blood utilized a standard salting-out method. The Agilent SureSelect Human All Exon Kit V6 was used to capture patients' DNA, followed by sequencing on an Illumina HiSeq 4000 machine. The sequencing achieved an average read depth exceeding 100x, with 98.0 % of the targeted genomic sequence having a depth of 20x or greater.

### 2.3 Variant filtering, annotation, and protein-protein interaction analysis

Following acquiring sequencing reads, we meticulously processed the data through a two-step approach. Initially, we trimmed the reads to eliminate adaptor sequences and masked regions exhibiting low complexity or low-quality sequencing. Subsequently, we executed the mapping of the reads to the hg19 whole genome utilizing the mem algorithm from the BWA aligner. For accurate variant calling, we adhered to the GATK Best Practices pipeline (https://gatk.broadinstitute.org/hc/en-us), employing the recommended tools. Finally, the identified variants underwent comprehensive annotation using the ANNOVAR tool. To further investigate the biological relevance of the identified variants, protein-protein interaction (PPI) analysis was conducted using the STRING database (https://string-db.org/).

### 2.4 Variant validation and Co-segregation analysis

Variants identified by WES were verified in the patients. Co-segregation analysis revealed the presence of these variants in affected patients and their healthy family members, consistent with an autosomal recessive inheritance pattern. Primer design using Primer 3 (primer sequences are available upon request), followed by verification using the ABI 3130 Genetic Analyzer machine, played a crucial role in confirming the identified variants.

## 3 Results

### 3.1 Genetic analysis and clinical data

In this study, twenty subjects, including 12 boys (60 %) and 8 girls (40 %) were evaluated (Table 1). The mean age of patients was 8.29 ± 2.14 years. Developmental delay (45 %) and movement dysfunction (30 %) were the most prevalent clinical manifestations; also, none of the patients had a familial history. The clinical, radiological and demographic information of patients was presented in Table 1.

**Table 1:** Demographic information of patients.

Variables		Results
Gender (%)	Boy	12 (60)
	Girl	8 (40)
Age, mean ± SD (year)		7.29 ± 2.14
Clinical findings (%)	Developmental delay	7 (45)
	Movement dysfunction	6 (35)
	Ataxia	3 (15)
	Hypomyelination	2 (10)
	Microcephaly	5 (25)
	Macrocephaly	1 (5)
Seizure type (%)	Focal	5 (25)
	Generalized	15 (75)
MRI findings	Atrophy signs	9 (45)
	White matter hyperintensity	8 (40)
	Corpus callosum abnormality	6 (30)
	Myelin abnormality	5 (25)

In total, 24 WES were performed. The overall diagnostic yield of WES in identifying genetic variants involved was 25 % (6 out of 24) (95 % confidence interval (CI): 14.8–31.3 %); by excluding parents and evaluating just patients with clinical findings, the diagnostic yield was 10 % (95 % CI: 12.3–31.3 %). Two homozygous autosomal recessive variants in two patients, alongside four heterozygous variants in their parents, were diagnosed.

**Fig. 1:**
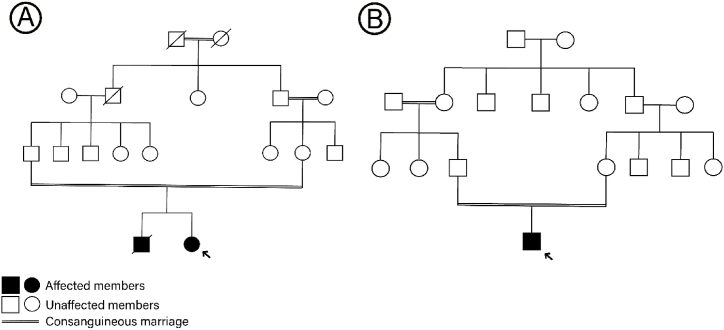
Pedigrees of the families under study (A and B). Males are represented by squares, and females by circles. Filled symbols denote affected individuals, and deceased individuals are indicated with a slash through the symbol. The proband is marked with an arrow for clarity.

Genetic analysis revealed significant variants in only two families (Fig. 1A and B). The comprehensive genetic analysis employed WES, leading to the identification and subsequent confirmation of variants via Sanger sequencing.

**Fig. 2:**
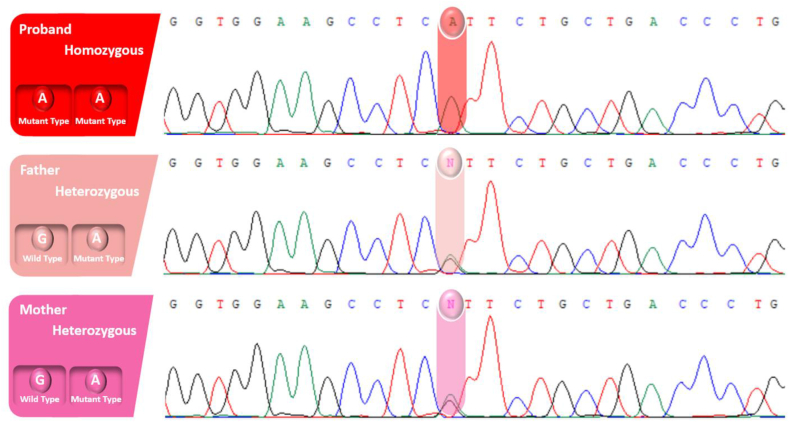
Illustrates the discovery of a variant in the AP3B2 gene and the genetic screening of the family under investigation. The direct sequencing of the patient's DNA uncovered a homozygous variant (c.3190G > A; p.Val1064Ile), while her unaffected parents carried the variant in a heterozygous state.

In the first family, a newly discovered variant was identified in the AP3B2 gene (NM_001278512.2: c.3190G > A; p.Val1064Ile), located within exon 27. This variant displayed homozygosity in the proband and heterozygosity in the parents (Fig. 2). The substitution of guanine (G) with adenine (A) at position 3190 resulted in an amino acid change from valine (Val) to isoleucine (Ile) at position 1064. The proband, a 3-year-old girl, presented with a history of seizures since the neonatal period, characterized by generalized tonic-clonic movements. Developmental delays and regression in motor, microcephaly, and cognitive skills were noted. Abnormal electroencephalogram (EEG) patterns supported the diagnosis of Epileptic Encephalopathy, Early Infantile, 48 (EE-48). Tragically, the family previously experienced the expired a boy child at 40 days of age with recurrent seizures similar to those observed in the affected girl. Though the deceased boy did not undergo genetic testing, the strikingly similar clinical presentation and the confirmed diagnosis of EE-48 in the affected girl raised strong suspicions of a potential genetic etiology in the sibling. This variant strongly suggests an association with EE-48, highlighting the potential clinical significance of this genetic variation.

**Fig. 3:**
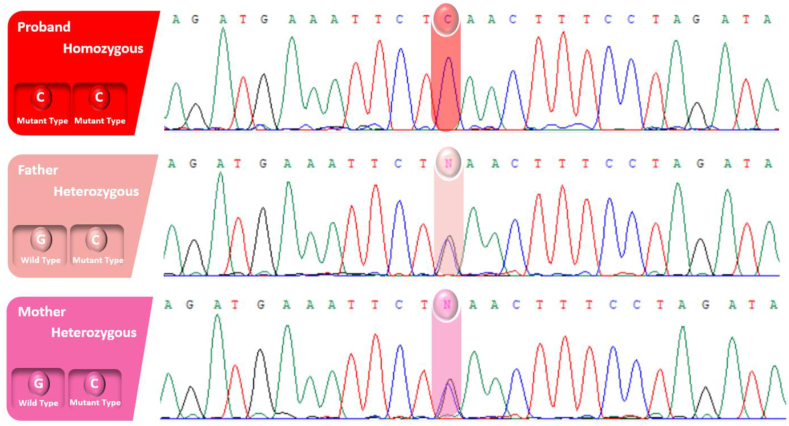
The direct sequencing of the patient's DNA uncovered a homozygous variant (c.1664G > C; p.Ter555Serext∗54) in the PIGB gene, while her unaffected parents carried the variant in a heterozygous state.

**Fig. 4:**
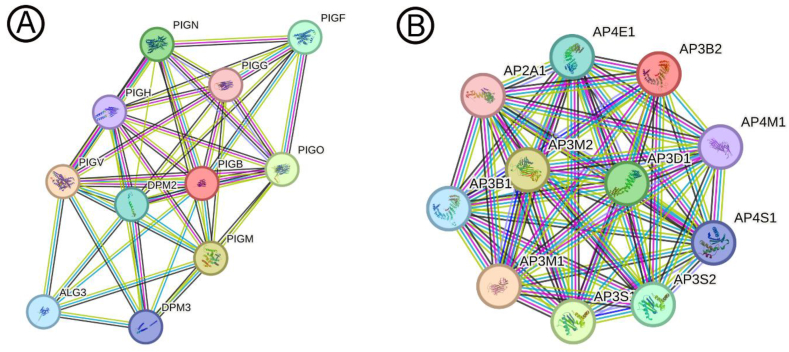
(A) PPI network of adapter protein complexes, including AP3B2 and related proteins. (B) PPI network of GPI anchor biosynthesis proteins, including PIGB and related proteins.

In the second family, an unreported variant was observed in the PIGB gene (NM_004855.5: c.1664G > C; p.Ter555Serext∗54), located in exon 12. Similar to the first case, this variant demonstrated homozygosity in the proband and heterozygosity in the parents (Fig. 3). The substitution of guanine (G) with cytosine (C) at position 1664 resulted in an amino acid alteration, transforming a termination codon (Ter) to serine (Ser) at position 555, along with an extended sequence. The proband, a 2-year-old boy, exhibited seizures in early infancy, characterized by generalized tonic-clonic movements evolving into a status epilepticus pattern. Developmental regression, profound intellectual disability, and abnormal EEG patterns consistent with epileptic encephalopathy were noted. A notable laboratory finding was a high serum alkaline phosphatase (ALP) level, measured at 820 U/L (normal range: 145–420 U/L for this age group). This elevated ALP level during the diagnostic workup suggests potential systemic involvement, emphasizing the association with Epileptic Encephalopathy, Early Infantile, 80 (EE-80). Conservation and functional impact analyses of the variant revealed modest conservation, as indicated by scores such as PhastCons100way (0.010) and PhyloP100way (−0.042), alongside PhyloP and PhastCons metrics for primates and vertebrates, which demonstrated varying levels of evolutionary conservation. Functional predictions, including fitCons (0.3192) and SiPhy29way logOdds (1.8968), further support the variant's potential pathogenicity, emphasizing its relevance in the observed clinical phenotype. Fig. 4A presents the protein-protein interaction (PPI) network of adapter protein complexes, including AP2A1, AP3B1, AP3B2, AP3D1, AP3M1, AP3M2, AP3S1, AP3S2, AP4E1, AP4M1, and AP4S1. Similarly, Fig. 4B displays the PPI network of glycosylphosphatidylinositol (GPI) anchor biosynthesis proteins, including PIGB, PIGG, PIGN, PIGM, PIGH, PIGF, PIGO, PIGV, DPM2, DPM3, and ALG3.

In contrast, genetic analysis in the remaining eighteen families revealed no variants associated with the observed clinical manifestations. The absence of detected genetic variants in these cases may suggest two possibilities: 1- genetic causes might lie in other genes not included in our panel or outside the scope of the current analysis; or 2- factors other than genetic variations, such as environmental or epigenetic influences, could contribute to the observed neurological disorders in these families.

## 4 Discussion

There is considerable literature about genetics and epilepsy. The findings revealed a strong correlation between phenotype-genotype in epileptic patients, which influences the treatment strategy [11].

Epilepsy is a brain disorder with a broad spectrum from minimal to severe seizures that can be accompanied by growth delay and cerebral palsy. On the other side, there is a specific phenotype of epilepsy that is attributed to specific genes, such as Dravet syndrome. These findings indicated a high heterogeneity among the genes involved in epilepsy, which indicates a need for genetic studies in this field. The present survey aims to investigate the gene defects in pediatrics with epilepsy. The incidence of idiopathic generalized epilepsy was reported at 2.9/100,000 in the general population, which is accompanied by a favorable response to treatment [12]. In this regard, in the present study, patients with idiopathic epilepsy were excluded.

Our findings revealed two novel variants in AP3B2 and PIGB genes. Adaptor proteins (AP) are five complexes (AP-1 to AP-5) and various isoforms that play a critical role in secretory and endocytic pathways [13,14]. There are 2 isoforms of AP-3: AP-3A (Ubiquitous) and AP-3B (neuron-specific). AP3 complex is required in clathrin-coated synaptic vesicle formation, which plays a crucial role in signal-mediated trafficking of integral membrane proteins [13]. The AP3 complex has various expression patterns based on the subunit's types. The beta subunit neuron is specifically encoded by AP3 and was identified for the first time in neurological disorders. Target cargo into vesicles assembled is vital in neural signal transmission [15]. The encoded subunit binds clathrin and is phosphorylated by a casein kinase-like protein, which mediates synaptic vesicle coat assembly [16]. AP3 complex is critical in delivering cell bodies into neurites and nerve terminals [16]. Hence, any gene alteration causes loss of function variants in AP3, disruption of neurotransmitter release, EOE development and encephalopathy (EE-48) [13,14].

AP3B2 gene defect is a rare autosomal recessive variant with developmental and epileptic encephalopathy. One of our patients was a 3-year-old girl with a novel variant in the AP3-B2 gene. The second child of this family was a 40-day boy with suspicion of epileptic encephalopathy, too. Dilber et al. in Turkey, identified two novel variants in the AP3B2 gene, including c.445_448delGCTA (p.Ala149Serfs∗34) and c.2978_2979delAT (p.Pro993Argfs∗5) [17]. The main clinical finding was early-onset infantile epileptic encephalopathy and dysfunctions in movement, eye, and speech [17].

Drug resistance epilepsy (DRE) occurs in 14.6–25 % of epileptic patients [18]. Several risk factors for DRE development include neurological deficiency, abnormal EEG, and gene polymorphisms such as ABCB1 [18]. Kocaaga et al. evaluated 45 patients with DRE. Their results showed 26 variants responsible for DRE, 19 of which were novel [19]. Among novel detected genes, a homozygous frameshift variant in AP3B2, c.2705_2706dup (p.Ile903ProfsTer36) in a generalized epileptic patient [19]. The clinical manifestations in patients who inherit autosomal recessive AP3B2 gene variants are EER, postnatal microcephaly, eye atrophy, and growth delay. Additional variants must be suspected in cases with AP3B2 gene defects with more clinical findings. In this line, Ueda et al. reported a case of Angelman syndrome with various features. The results of WES demonstrated two types of autosomal recessive variants, including HERC2 and AP3B2 [20]. They concluded that unusual features are due to variant combination [20]. In addition to the AP3B2 gene abnormalities, the AP3B2 autoantibody causes cerebral ataxia and cognitive impairment [21]. Also, AP3B2 autoantibody may be produced following traumatic brain injury [22].

Among our patients, the PIGB was identified, too. The patient was a 2-year-old boy with similar features to the previous subject, including EER, development delay, and intellectual delay. The result of WES showed a novel variant in PIGB dene. PIGB belongs to glycosylphosphatidylinositol (GPI), which is critical in neurogenesis and development. As expected, the ALP of the patient was increased, which is characteristic of GPI gene defect. GPI is a vital family of membrane-binding proteins that add anchors undergoing remodeling and modifications according to their role and cell. Any changes in the assembling GPI result in inherited GPI deficiencies [23]. In neurogenesis, GPI-anchored proteins play a vital role in synaptic adhesion pathways [24]. Our data indicated the EER due to PIGB. In this line, the results of Efthymiou et al. indicated that biallelic variants of PIGS variants cause epilepsy development in response to treatment with pyridoxine vitamin [25]. Replacement of highly conserved Leucine with Phenylalanine (p.L378F) in PIGO causes development clinical findings such as epileptic encephalopathy, developmental delay, hypotonia, ataxia, and hyperphosphatemia; this is a novel variant that was reported by Aguech et al. [26]. The influence of PIGB gene defects in epilepsy development is well understood. However, the exact involved pathophysiology mechanisms of GIP in epilepsy remained unclear [27]. In PIGB variants, the functions of inhibitory neurons are delayed with structural disruption in length, leading and trailing processes [28]. Additionally, any PIGB variants are accompanied by a reduction of inhibitory GABAergic interneurons, In severe phenotype of epilepsy due to PIGB variants, DOORS syndrome may be observed [29]. Hypomyelination was detected in our patient, while the result of Schiavoni et al. reported a similar case with our clinical features with different missense variant of PIGB, NM_004855.4: c.463G > C, p. (Asp155His) [30].

In our investigation, the overall diagnostic yield of WES in identifying causative genetic variants was 25 %. In similar studies, the reported diagnostic yield of WES in epilepsy varies from 8 to 37 % [6,31]. The extended exome analysis increases 4.2 % the diagnostic yield of heterogenous disorders [32]. Also, using specific selection criteria can improve the diagnostic yield of WES up to 80 % [33]. It can be concluded that the selection criteria directly influenced the diagnostic yield of WES.

The PPI networks presented in Fig. 3A and B provide crucial insights into the molecular mechanisms underlying the pathogenesis of EOE in the studied families. Fig. 4A highlights the PPI network of adapter protein complexes, including AP2A1, AP3B1, AP3B2, AP3D1, AP3M1, AP3M2, AP3S1, AP3S2, AP4E1, AP4M1, and AP4S1. Among these, AP3B2 is particularly noteworthy due to its established role in intracellular vesicle trafficking and synaptic function. The AP3B2 variant (NM_001278512.2: c.3190G > A; p.Val1064Ile) identified in the first family likely disrupts these processes, which are critical for maintaining neuronal homeostasis. The network demonstrates the extensive interconnectivity of AP3B2 with other adapter proteins, such as AP3B1, AP3D1, and AP3M1, suggesting that the identified mutation may compromise not only vesicle-mediated transport but also downstream cellular functions essential for neurodevelopment. This disruption aligns with the clinical phenotype of EE-48, characterized by early-onset seizures, developmental delays, and severe neurocognitive impairments observed in the proband [13,16,34]. Similarly, Fig. 4B presents the PPI network of glycosylphosphatidylinositol (GPI) anchor biosynthesis proteins, including PIGB, PIGG, PIGN, PIGM, PIGH, PIGF, PIGO, PIGV, DPM2, DPM3, and ALG3. The PIGB variant (NM_004855.5: c.1664G > C; p.Ter555Serext∗54) detected in the second family introduces a truncating mutation, likely impairing PIGB's enzymatic role in the GPI-anchor synthesis pathway. This pathway is crucial for anchoring proteins to the cell membrane, a process vital for neuronal signaling, adhesion, and synaptic stability. The network demonstrates that PIGB's interactions with key proteins, including PIGG, PIGN, PIGM, and PIGO, are integral to maintaining the structural and functional integrity of the GPI biosynthesis pathway. Disruption of these interactions could result in widespread cellular dysfunction, consistent with the severe clinical features of EE-80 observed in the proband, including early-onset seizures, profound developmental regression, and systemic involvement, as suggested by elevated serum alkaline phosphatase levels. The dense interconnectivity observed in both PPI networks underscores the centrality of AP3B2 and PIGB within their respective pathways, suggesting that mutations in these genes may have cascading effects on cellular function. Moreover, the inclusion of other critical interacting proteins in these networks, such as DPM2, DPM3, and ALG3 in the GPI biosynthesis network, highlights the broader implications of these mutations. These findings reinforce the importance of PPI network analysis in understanding the molecular mechanisms of disease and suggest that disruptions in vesicle trafficking and GPI-anchor biosynthesis are key contributors to the pathophysiology of EOE [29,[35], [36], [37]]. Collectively, this study elucidates the molecular basis of the identified genetic variants and their association with distinct yet overlapping phenotypes of epileptic encephalopathy, offering a foundation for future research into targeted therapeutic approaches.

While our study provides valuable insights into the genetic underpinnings of EEO, it is important to acknowledge its limitations. The relatively small sample size reduces the statistical power to detect less common genetic variants. Additionally, the scope of our analysis was restricted to exonic regions within a predefined panel of genes, which means potential pathogenic variants in other genes or non-coding regions may have been missed. Future research should include a broader analysis of genes and utilize comprehensive approaches, such as whole-genome sequencing (WGS), to capture variants outside the current panel. Moreover, integrating functional assays and multi-omics approaches could enhance our understanding of the molecular mechanisms involved. Addressing these limitations will pave the way for more holistic insights into the complex etiologies of epilepsy and related neurological disorders.

## 5 Conclusion

This study underscores the value of WES in uncovering genetic variants associated with epilepsy, focusing on two novel findings: AP3B2 (NM_001278512.2: c.3190G > A; p.Val1064Ile) and PIGB (NM_004855.5: c.1664G > C; p.Ter555Serext∗54). These variants provide critical insights into epilepsy pathophysiology, with the PIGB variant notably linked to elevated ALP levels, suggesting a potential biomarker for further investigation. By prioritizing detailed analysis of impactful variants, this study not only advances understanding of the genetic basis of epilepsy but also highlights the clinical significance of WES in improving diagnosis and management. Identifying actionable genetic variants enables early diagnosis, personalized treatment, and better patient outcomes, offering a pathway toward more precise and effective strategies for addressing complex neurological disorders.

## CRediT authorship contribution statement

**Pooyan Alizadeh:** Validation, Project administration, Investigation. **Armin Jahangiri Babadi:** Writing – original draft, Methodology, Data curation. **Nemat Ghadiri:** Resources, Investigation, Formal analysis. **Mostafa Neissi:** Visualization, Software, Formal analysis. **Masoud Zeinali:** Writing – review & editing, Writing – original draft, Supervision, Conceptualization.

## Declaration of generative AI in scientific writing

The authors declare that the AI was not used in any manuscript preparation stage.

## Declaration of competing interest

The authors declare that they have no known competing financial interests or personal relationships that could have appeared to influence the work reported in this paper.

## Data Availability

No data was used for the research described in the article.
